# The efficacy of ileostomy after laparoscopic rectal cancer surgery: a meta-analysis

**DOI:** 10.1186/s12957-021-02432-x

**Published:** 2021-11-04

**Authors:** Yu Mu, Linxian Zhao, Hongyu He, Huimin Zhao, Jiannan Li

**Affiliations:** 1 Department of General Surgery, The Second Hospital of Jilin University, Changchun, 130041 Jilin China; 2 Operating Theater and Department of Anesthesiology, The Second Hospital of Jilin University, Changchun, 130041 Jilin China

**Keywords:** Clinically significant anastomotic leakage, Ileostomy, Laparoscopy, Rectal cancer

## Abstract

**Background:**

Protective ileostomy is always applied to avoid clinically significant anastomotic leakage and other postoperative complications for patients receiving laparoscopic rectal cancer surgery. However, whether it is necessary to perform the ileostomy is still controversial. This meta-analysis aims to analyze the efficacy of ileostomy on laparoscopic rectal cancer surgery.

**Methods:**

Cochrane Library, EMBASE, Web of Science, and PubMed were applied for systematic search of all relevant literature, updated to May 07, 2021. Studies compared patients with and without ileostomy for laparoscopic rectal cancer surgery. We applied Review Manager software to perform this meta-analysis. The quality of the non-randomized controlled trials was assessed using the Newcastle-Ottawa scale (NOS), and the randomized studies were assessed using the Jadad scale.

**Results:**

We collected a total of 1203 references, and seven studies were included using the research methods. The clinically significant anastomotic leakage rate was significantly lower in ileostomy group (27/567, 4.76%) than that in non-ileostomy group (54/525, 10.29%) (RR = 0.47, 95% CI 0.30–0.73, *P* for overall effect = 0.0009, *P* for heterogeneity = 0.18, *I*^2^ = 32%). However, the postoperative hospital stay, reoperation, wound infection, and operation time showed no significant difference between the ileostomy and non-ileostomy groups.

**Conclusion:**

The results demonstrated that protective ileostomy could decrease the clinically significant anastomotic leakage rate for patients undergoing laparoscopic rectal cancer surgery. However, ileostomy has no effect on postoperative hospital stay, reoperation, wound infection, and operation time. The efficacy of ileostomy after laparoscopic rectal cancer surgery: a meta-analysis.

**Supplementary Information:**

The online version contains supplementary material available at 10.1186/s12957-021-02432-x.

## Introduction

Colorectal cancer (CRC) ranks third in the global incidence of malignant tumors, affecting human health seriously [1]. According to the American Cancer Society statistics, the incidence of CRC in males is lower than that in prostate and lung cancer. In females, CRC incidence is lower than that in breast and lung cancer [2]. CRC ranks second in males and third in females worldwide [2].

Since the 1990s, laparoscopic surgery has attracted more and more attention as a new minimally invasive surgical method to treat many diseases [3, 4]. Many multi-center researchers and a large number of randomized controlled trials have confirmed the safety and reliability of laparoscopic rectal cancer surgery [5–7]. The laparoscopic operation can follow the total mesorectal excision (TME) principle with many advantages, including minor surgical trauma and bleeding, lower surgical complications and postoperative pain, and faster postoperative recovery to achieve R0 resection of the tumor [8, 9]. As a result, laparoscopic low anterior resection (LAR) has become an essential operation for rectal cancer.

Many patients receiving rectal cancer surgery routinely undergo protective ileostomy to avoid clinically significant anastomotic leakage and other complications. However, whether it is necessary to perform the ileostomy is still controversial. At present, there are only a small number of randomized controlled trials and comparative studies assessing the effect of ileostomy during laparoscopic rectal cancer surgery. Therefore, the efficacy of ileostomy on laparoscopic rectal cancer surgery was analyzed using the meta-analysis.

## Methods

### Search strategies

We perform this study according to the stated guidelines of Preferred Reporting Items for Systematic Reviews and Meta-Analysis (PRISMA) [10]. The protocol was registered with PROSPERO in advance (CRD42021253824) (https://www.crd.york.ac.uk/PROSPERO/#recordDetails). Cochrane Library, EMBASE, Web of Science, and PubMed were applied for systematic search of all relevant literature, updated to May 07, 2021, using the search strategy including the terms for “Rectum tumor,” “Rectal Neoplasms,” “Ileostomy,” “Ileostomies,” “Laparoscopy,” “Celioscopy,” and their variants. The detailed search strategy was shown in Additional file 1. In addition, reference lists of all primary studies and systematic reviews published were searched manually for additional trials.

### Inclusion and exclusion criteria

The inclusion criteria include [1] patients who were diagnosed with rectal cancer by histopathology and were treated with laparoscopic rectal cancer surgery, [2] clinical trials compared patients with and without protective ileostomy, [3] patients were grouped by whether they received protective ileostomy [4] at least one of the following outcomes were reported (incidence of postoperative anastomotic fistula, reoperation rate, length of hospital stay, incidence of postoperative peritonitis, and postoperative incision infection rate), and [5] studies reported in English.

The exclusion criteria include [1] studies included benign colorectal diseases [2]; review, letter, case report, or meta-analysis [3]; other surgical methods were used, such as open abdominal Dixon surgery, Miles, robot-assisted resection of the rectum, and natural orifice transluminal endoscopic surgery (NOTES) [4]; single group studies; and [5] complete extraction could not be obtained.

In the case of studies comprising the same patient cohort, we only selected the most recent or the complete study.

### Data extraction and methodology quality assessment

MY, ZHM, ZLX, HHY, and LJN extracted and assessed the data independently. At least two researchers assessed each citation. The extracted data mainly included the first author, publication year, number of patients, age, sex, clinical characteristics, and outcomes. The primary outcome mainly included clinically significant anastomotic leakage rate, and the secondary outcomes include operation time, length of hospital stay, complication rate, and reoperation rate. Any uncertainties about the extracted data were carefully discussed until a consensus was reached.

The NOS was applied for the assessment of non-randomized controlled trials in this study, including selecting patients, comparability and controls on the study design, and outcome assessment. The study was considered moderate to high quality if the score was more than six. Randomized studies were assessed with the Jadad scale. Two researchers scored each study independently, and a discussion was made if there was a disagreement.

### Statistical analysis

Review Manager 5.3 provided by the Cochrane Collaboration Network was used for statistical analysis. Weighted mean differences (MDs) and risk ratios (RRs) with 95% confidence intervals (CI) were calculated for analyzing continuous and dichotomous data, respectively. *P* < 0.05 was considered as statistical significance. *I*^2^ test was applied to assess the heterogeneity of the studies. There was significant heterogeneity when *I*^2^ > 50% or *P* < 0.05 and the random-effects model should be used. Otherwise, the fixed-effects model should be applied. We would employ a sensitivity analysis when there was significant heterogeneity of studies. By omitting each study at a time, the effect of an individual study on the overall heterogeneity could be analyzed. Funnel plots assessed publication bias.

## Results

### Study characteristics

**Fig. 1:**
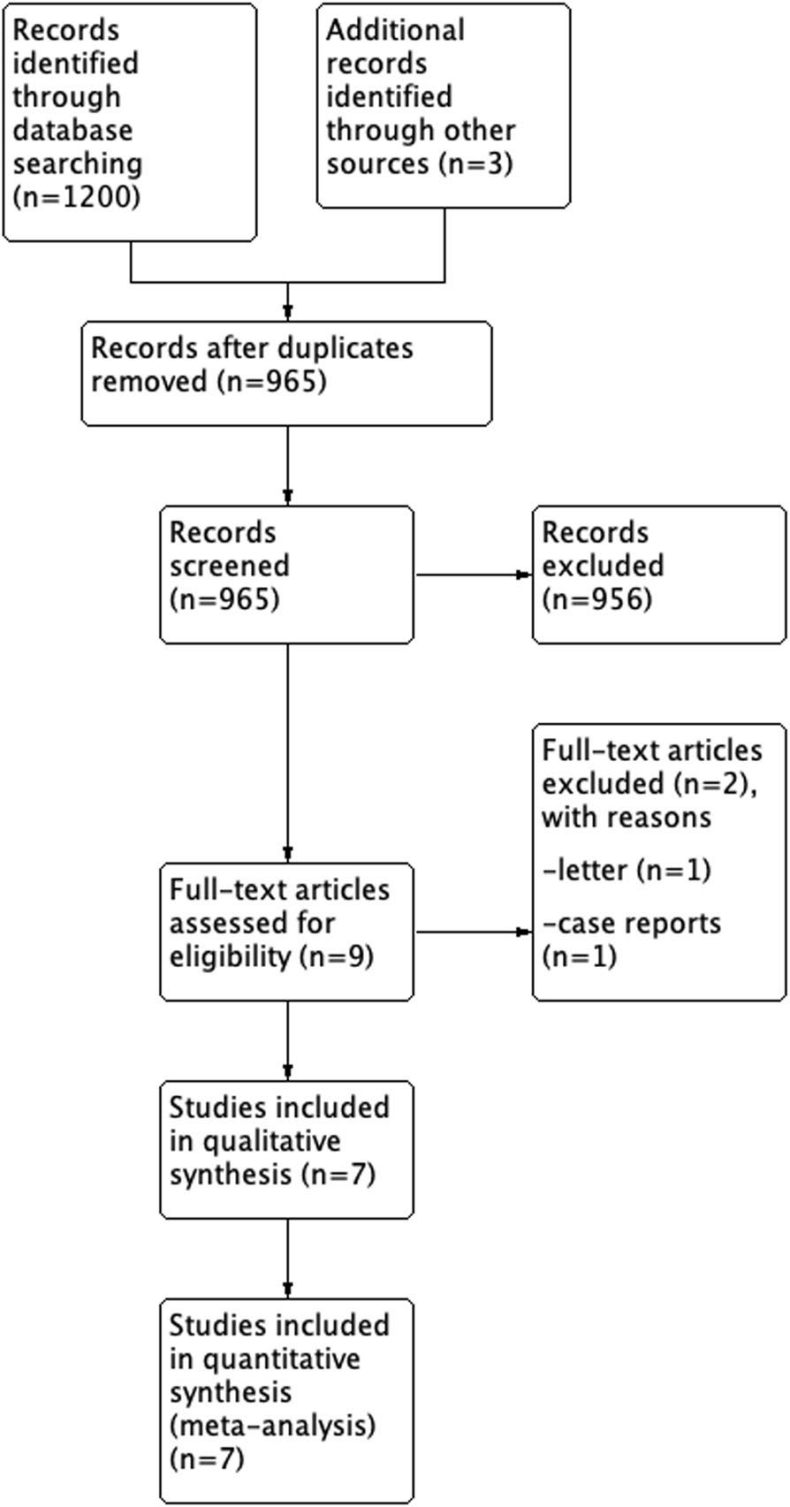
Flowchart of the included studies

According to the research methods, there were a total of 1203 references in this study, including 15 references in Cochrane Library, 650 references in EMBASE, 403 references in Web of Science, 132 references in PubMed, and other three additional references identified through manual reference searching. The 238 duplicated citations were deleted, and 952 studies were excluded after scanning the title and abstract of the remaining citations according to the inclusion and exclusion criteria. Finally, seven cohort studies were chosen in this meta-analysis [11–17], and only one was a randomized controlled trial (RCT), and others were comparative. Figure 1 shows the flow diagram of the included studies.

There were 1092 patients in this meta-analysis, with 567 patients in the ileostomy group and 525 patients in the non-ileostomy group, with an average patient age of 61.5 years in the ileostomy group and 62.6 in the no ileostomy group. All patients had been confirmed as rectal cancer by postoperative pathologic diagnosis and met the standard of laparoscopic rectal cancer surgery. We collected the characteristics of all the seven included cohort studies in Table 1, where six comparative studies scored six or more assessed by NOS, and one RCTs scored three assessed with the Jadad scale.

**Table 1:** Basic characters of literatures

Study		Karahasanoglu	Skrovina	Huang	Mari	Ihnat	Zanguie	Shimizu
Year		2011	2011	2014	2015	2016	2018	2020
Country		Turkey	Slovakia	China	Italy	Czech	Iran	Japan
NOS/JADAD		6	7	6	3	8	6	6
Type		CS	CS	CS	RCT	CS	CS	CS
Patients (*N*)	Ileostomy	23	50	60	55	78	75	226
	No ileostomy	54	64	60	52	73	31	191
Age (mean)	Ileostomy	59	63	61	71	63	52	62
	No ileostomy	61	63	61	69	64		63
Male	Ileostomy	15	36	34	26	50	92	174
	No lleostomy	25	37	34	27	28		101
Female	Ileostomy	8	14	26	29	43	63	52
	No lleostomy	29	27	26	25	30		90
Anastomotic leakage	Ileostomy	0	2	0	3	2	1	19
	No lleostomy	3	11	6	4	7	4	19
Postoperative hospital stay (day)	Ileostomy	NA	NA	7.4±1.1	6.3±1.6	11.3±8.5	NA	13.5±12.4
	No lleostomy			13.2±2.5	6.6±1.7	8.1±6.9		10.8±12.5
Reoperation	Ileostomy	NA	1	NA	1	NA	NA	1
	No lleostomy		6		3			0
Wound infection	Ileostomy	2	NA	1	2	NA	NA	NA
	No lleostomy	1		2	3			
Operation time (min)	Ileostomy	NA	NA	156.3±10.6	185±32	NA	NA	263±65.6
	No lleostomy			155.3±11.7	186±47			220.4±60.1

*NOS* Newcastle-Ottawa scale, *JADAD* Jadad scale, *CS* cohort studies, *RCT* randomized controlled trials, *NA* not applicable

### Pooled analysis

#### Clinically significant anastomotic leakage

**Fig. 2:**
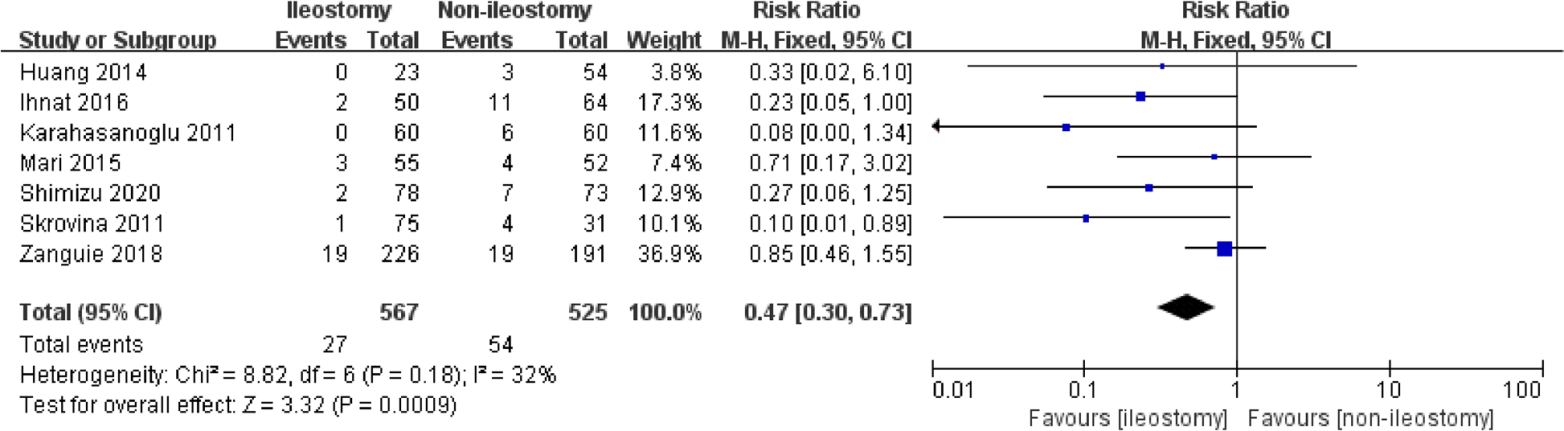
The forest map for clinically significant anastomotic leakage

**Fig. 3:**
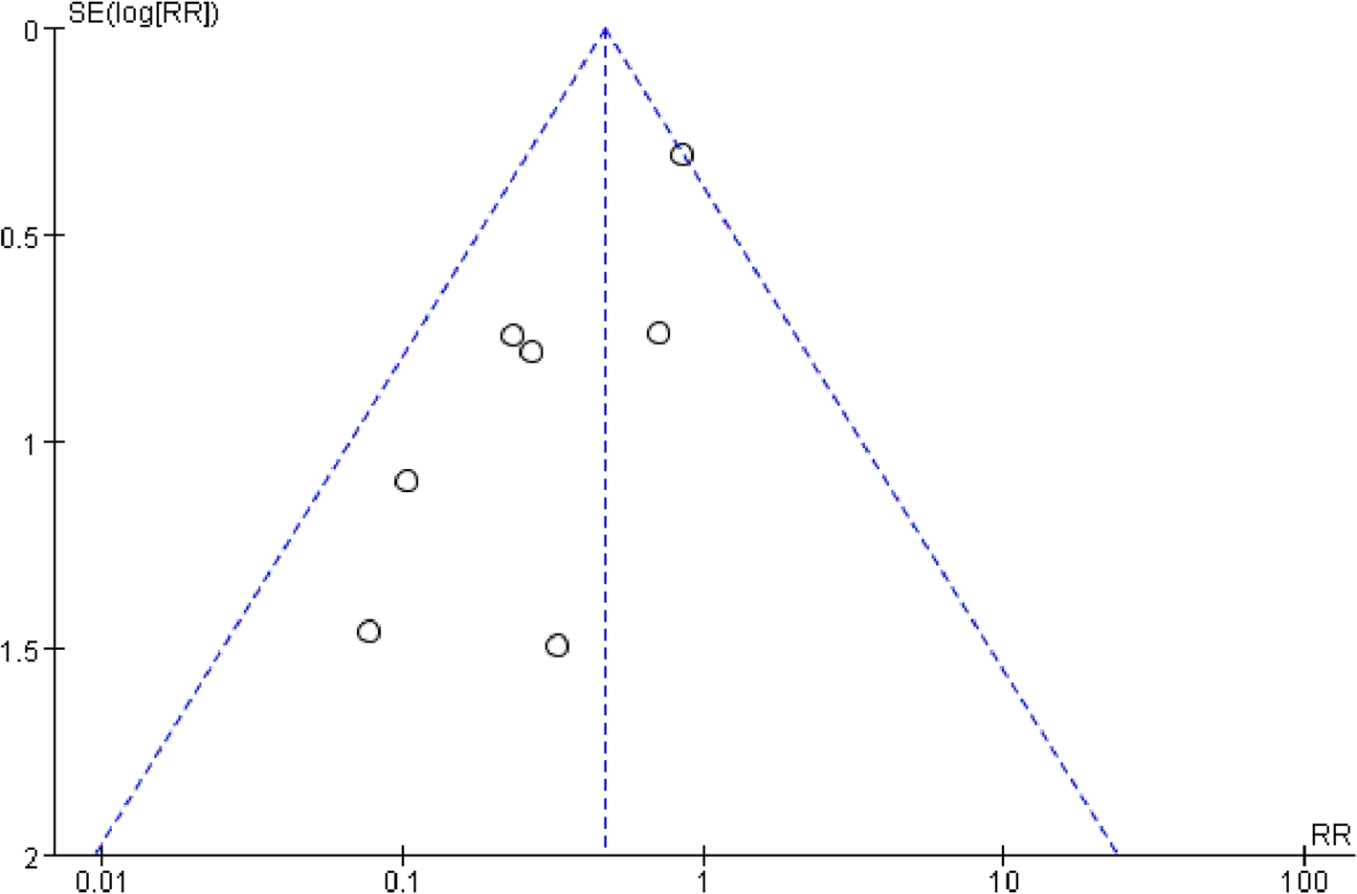
Funnel plot of clinically significant anastomotic leakage

All seven studies involved the clinically significant anastomotic leakage data. Figure 2 shows significant difference in the clinically significant anastomotic leakage rate between the ileostomy group (27/567, 4.76%) and non-ileostomy group (54/525, 10.29%) (RR = 0.47, 95% CI 0.30–0.73, *P* for overall effect = 0.0009, *P* for heterogeneity = 0.18, *I*^2^ = 32%). The funnel plot on clinically significant anastomotic leakage shows that all the studies lie inside the limit of 95% CI, indicating no obvious publication bias (Fig. 3).

#### Postoperative hospital stay

**Fig. 4:**
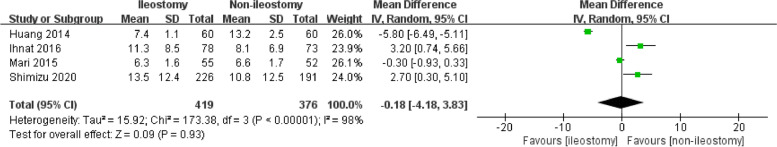
The forest map for postoperative hospital stay

Four studies reported the postoperative hospital stay with 419 patients in the ileostomy group and 376 patients in the non-ileostomy group. There is no statistical difference between the two groups (Fig. 4, MD = −0.18, 95% CI −4.18–3.83, *P* for overall effect = 0.93, *P* for heterogeneity < 0.00001, *I*^2^ = 98%).

#### Reoperation

**Fig. 5:**
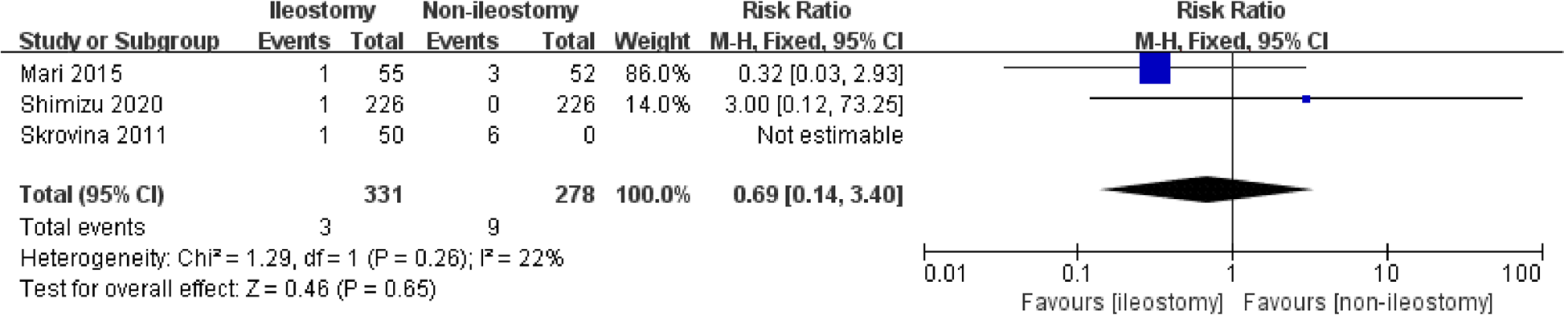
The forest map for reoperation after laparoscopic rectal cancer surgery

The reoperation outcome in all included studies was specifically caused by anastomotic leakage. Three studies reported the reoperation outcome with 331 patients in the ileostomy group and 278 patients in the non-ileostomy group. There is no statistical difference between the two groups (Fig. 5, RR = 0.69, 95% CI 0.14–3.40, *P* for overall effect = 0.65, *P* for heterogeneity = 0.26, *I*^2^ = 22%).

#### Wound infection

**Fig. 6:**
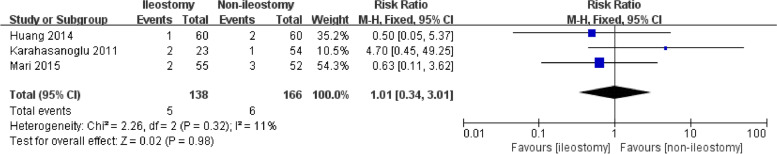
The forest map for wound infection after surgery

Three studies reported the data on wound infection. The analysis shows no difference in wound infection rate between ileostomy group (5/138, 3.62%) and non-ileostomy group (6/166, 3.61%) (Fig. 6, RR = 1.01, 95% CI 0.34–3.01, *P* for overall effect = 0.98, *P* for heterogeneity < 0.32, *I*^2^ = 11%).

#### Operation time

**Fig. 7:**
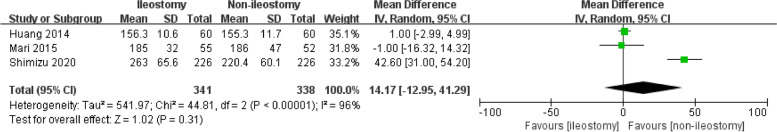
The forest map for operation time

Three studies reported the operation time of 341 patients in the ileostomy group and 338 patients in the non-ileostomy group. There was significant heterogeneity among the studies, and a random-effects model was used for meta-analysis. Data analysis shows no statistical difference between the two groups (Fig. 7, MD = 14.17, 95% CI −12.95–41.29, *P* for overall effect = 0.31, *P* for heterogeneity < 0.00001, *I*^2^ = 96%).

### Sensitivity analysis

There was no significant heterogeneity in the clinically significant anastomotic leakage, reoperation after laparoscopic rectal cancer surgery, and wound infection after surgery. As a result, we performed the fixed-effects model analysis for these three models. Significant heterogeneity was observed in the postoperative hospital stay and operation time. Therefore, the random-effects model was performed for the two models. Unfortunately, the sensitivity analysis did not reduce heterogeneity in the postoperative hospital stay and operation time. After removing the study of Shimizu et al., no significant heterogeneity was observed in the operation time. However, during the sensitivity analysis, the pooled results of postoperative hospital stay and operation time remained unchanged. We analyze that the heterogeneity may be due to the regional differences and medical level. For example, the laparoscopic rectal cancer surgery regimens have changed significantly, and postoperative treatment regimens have varied a lot over the past 10 years.

## Discussion

Colorectal malignancies are common digestive system tumors with increased morbidity and mortality every year [1]. Surgical resection is still the main method to treat CRC. TME is the key to radical resection for rectal cancer [4, 8, 18], and rectal washout has been integrated with the TME technique in recent years [19]. According to the principles of TME, laparoscopic LAR for rectal cancer has become the mainstream treatment method for patients [20, 21]. Mainly, laparoscopic tumor-specific mesorectal excision (TSME) has been utilized for upper rectal cancer [22]. Many problems that cannot be solved by conventional laparotomy, such as anus-preserving surgery for rectal cancer with anal distance less than 5cm, can be solved by laparoscopic technique, which significantly improves the life quality of patients [23, 24]. Laparoscopic surgery has many advantages, such as a clear vision of operation, small incision, mild postoperative pain, and short hospital stay [25–27]. Especially, to pursue better treatment benefits, many laparoscopic surgery regimens have been improved, such as 3D laparoscopy, robotic surgery, single-incision laparoscopic surgery, two-port laparoscopic anterior resection, and LAR with natural orifice specimen extraction (NOSE-LAR) [28–32]. Moreover, some researchers proposed that sphincter-saving rectal resections can be seen as a feasible alternative to extra levator abdominoperineal resection or transanal total mesorectal excision [33].

Clinically significant anastomotic leakage is the main postoperative complication that threatens the life of patients with rectal cancer. The CT scan was the most common diagnostic method, and contrast enema, endoscopic examination, reoperation, and other postoperative complications also can assist in the diagnosis [34]. Clinically significant anastomotic leakage for laparoscopic rectal cancer surgery usually occurs on the 7th day post-surgery [35]. Generally, the small anastomotic leakage can be treated through conservative treatment (fasting water, nutritional support, anti-inflammation, fluid replacement), while the severe clinically significant anastomotic leakage requires surgical intervention [36]. We mainly focused on whether protective ileostomy can decrease the rate of clinically significant anastomotic leak, and tiny leaks are not evaluated in any studies.

Protective colostomy or ileostomy has been performed to reduce the incidence of clinically significant anastomotic leakage. However, it is still controversial that whether it is beneficial to perform the protective stoma. Some surgeons believe that preventive intestinal stoma is unnecessary because most clinically significant anastomotic leakage can be cured conservatively, and intestinal stoma increases the cost of hospitalization and requires secondary surgery [37–39]. The second surgery will undoubtedly delay the subsequent treatment of patients with rectal cancer surgery, especially those old and have many underlying diseases (such as postoperative chemoradiotherapy), and even accelerate the deterioration and increase mortality [40–43]. On the other hand, frail patients often require ileostomy closure surgery before adjuvant therapy (due to frequent nutritional deficiencies), which delays adjuvant therapy.

Moreover, researchers have not agreed on the concrete interval time from ileostomy closure surgery to adjuvant therapy. Some researchers believe that protective intestinal stoma can reduce intestinal edema, promote local intestinal function recovery, facilitate anastomotic healing, and reduce clinically significant anastomotic leakage and other complications [11]. This meta-analysis was based on seven comparative studies with more than 1000 patients. The results showed that protective ileostomy could reduce clinically significant anastomotic leakage in patients who underwent laparoscopic rectal cancer surgery. However, ileostomy has no influence on postoperative hospital stay, reoperation, wound infection, and operation time.

According to the principles of enhanced recovery after surgery (ERAS), postoperative recovery has been significantly increased [44]. The postoperative hospital stay showed no difference in the analyzed groups. According to the combination of included studies and clinical experience, although preventive stoma could reduce clinically significant anastomotic leakage, most clinically significant anastomotic leakage could be cured through conservative treatment. There was no significant difference in the perioperative reoperation rate between the two groups of patients. Besides, there was no statistical difference in wound infection rate and operation time in the ileostomy and non-ileostomy groups.

There are some limitations to this meta-analysis. First of all, the funnel plot suggests that publication bias has existed in this study because the included studies are all retrospective studies without a random controlled trial. Second, the surgeons have a clinical bias in performing protective ileostomy for patients receiving laparoscopic rectal cancer surgery possibly. Many different factors can influence the clinically significant anastomotic leakage rate, such as anastomotic position, sex, tumor diameter, operation time, location of ligation IMA, and postoperative management [36, 45, 46]. Therefore, there is bias in choosing ileostomy for patients receiving laparoscopic rectal cancer surgery. Third, the bias is caused by the length between the anastomosis and anal verge, not reported in all studies. It seems that clinically significant anastomotic leakage is tentative to occur when the anastomosis is lower [47, 48]. Fourth, the types of performed rectal resection are not thoroughly evaluated. In four studies, the authors have reported them as LAR, while in the other three studies, it has been TME. The other two studies have not reported rectal resection type. However, concerning the relative publication date of all seven studies, LAR is performed according to the principles of TME in all the cases. In addition, because it is unclear whether these included patients have received neoadjuvant therapy or not, this article did not evaluate the influence of neoadjuvant therapy on the leakage rate. Interestingly, a meta-analysis has reported that neoadjuvant therapy did not increase the rate of postoperative anastomotic leakage after middle and low rectal anterior resection [49], which guides future research. Finally, this study did not analyze whether ileostomy can affect the long-term survival rate of patients, bowel function, and postoperative quality of life.

## Conclusion

The study indicates that protective ileostomy can decrease the clinically significant anastomotic leakage rate in laparoscopic rectal cancer surgery patients. However, ileostomy has no effects on postoperative hospital stay, reoperation, and wound.

## Supplementary Information

**Additional file 1: Appendix 1**. The detailed search strategies.

## Data Availability

The datasets supporting the conclusions of this article are included within the article.

## Abbreviations

NOS: Newcastle-Ottawa scale
CRC: Colorectal cancer
TME: Total mesorectal excision
LAR: Low anterior resection
NOTES: Natural orifice transluminal endoscopic surgery
MDs: Mean differences
RRs: Risk ratios
CI: Confidence intervals
RCTs: Randomized controlled trials
TSME: Tumor-specific mesorectal excision
NOSE-LAR: LAR with natural orifice specimen extraction
ERAS: Enhanced recovery after surgery
IMA: Inferior mesenteric artery
