# Supplementary material for: The efficacy of ileostomy after laparoscopic rectal cancer surgery: a meta-analysis
Source: World J Surg Oncol. 2021 Nov 4;19:318. doi: 10.1186/s12957-021-02432-x (PMC8567543; doi:10.1186/s12957-021-02432-x)
Supplement: Supplementary file 1 — Additional file 1: Appendix 1. The detailed search strategies. [file 12957_2021_2432_MOESM1_ESM.docx]

The efficacy of ileostomy after laparoscopic rectal cancer surgery: a meta-analysis.

Yu Mu^1,#^, Linxian Zhao^1,#^, Hongyu He^2^, Huimin Zhao^2^, Jiannan Li^1,^*

^1^Department of General Surgery, The Second Hospital of Jilin University, Changchun Jilin 130041, China

^2^Operating Theater and Department of Anesthesiology, The Second Hospital of Jilin University, Changchun Jilin 130041, China

Yu Mu^#^ and Linxian Zhao^#^ contributed equally to this work.

Correspondence to:

Jiannan Li, Department of General Surgery, The Second Hospital of Jilin University, Changchun Jilin 130041, China. E-mail: jnli@ciac.ac.cn

**Appendix 1. The detailed search strategies**

**1. The terms of Cochrane Library**

#1 MeSH descriptor: [Rectal Neoplasms] explode all trees

#2 ("rectal neoplasms"):ti,ab,kw OR ("neoplasm, rectal"):ti,ab,kw OR ("rectal neoplasm"):ti,ab,kw OR ("rectum neoplasms"):ti,ab,kw OR ("neoplasm, rectum"):ti,ab,kw OR ("rectum neoplasm"):ti,ab,kw OR ("rectal tumors"):ti,ab,kw OR ("rectal tumor"):ti,ab,kw OR ("tumor, rectal"):ti,ab,kw OR ("neoplasms, rectal"):ti,ab,kw OR ("cancer of rectum"):ti,ab,kw OR ("rectum cancers"):ti,ab,kw OR ("rectal cancer"):ti,ab,kw OR ("cancer, rectal"):ti,ab,kw OR ("rectal cancers"):ti,ab,kw OR ("rectum cancer"):ti,ab,kw OR ("cancer, rectum"):ti,ab,kw OR ("cancer of the rectum"):ti,ab,kw

#3 (#1) OR (#2)

#4 MeSH descriptor: [Ileostomy] explode all trees

#5 ("ileostomy"):ti,ab,kw OR ("ileostomies"):ti,ab,kw OR ("tube ileostomy"):ti,ab,kw OR ("ileostomies, tube"):ti,ab,kw OR ("ileostomy, tube"):ti,ab,kw OR ("tube ileostomies"):ti,ab,kw OR ("incontinent ileostomy"):ti,ab,kw OR ("ileostomies, incontinent"):ti,ab,kw OR ("ileostomy, incontinent"):ti,ab,kw OR ("incontinent ileostomies"):ti,ab,kw OR ("loop ileostomy"):ti,ab,kw OR ("ileostomies, loop"):ti,ab,kw OR ("ileostomy, loop"):ti,ab,kw OR ("loop ileostomies"):ti,ab,kw OR ("continent ileostomy"):ti,ab,kw OR ("continent ileostomies"):ti,ab,kw OR ("ileostomies, continent"):ti,ab,kw OR ("ileostomy, continent"):ti,ab,kw

#6 (#4) OR (#5)

#7 MeSH descriptor: [Laparoscopy] explode all trees

#8 ("laparoscopy"):ti,ab,kw OR ("laparoscopies) :ti,ab,kw OR ("celioscopy"):ti,ab,kw OR ("celioscopies"):ti,ab,kw OR ("peritoneoscopy"):ti,ab,kw OR ("peritoneoscopies"):ti,ab,kw OR ("surgical procedures, laparoscopic"):ti,ab,kw OR ("laparoscopic surgical procedure"):ti,ab,kw OR ("procedure, laparoscopic surgical"):ti,ab,kw OR ("procedures, laparoscopic surgical"):ti,ab,kw OR ("surgery, laparoscopic"):ti,ab,kw OR ("laparoscopic surgical procedures"):ti,ab,kw OR ("laparoscopic surgery"):ti,ab,kw OR ("laparoscopic surgeries"):ti,ab,kw OR ("surgeries, laparoscopic"):ti,ab,kw OR ("laparoscopic-assisted surgery"):ti,ab,kw OR ("laparoscopic-assisted surgeries"):ti,ab,kw OR ("surgeries, laparoscopic-assisted"):ti,ab,kw OR ("surgery, laparoscopic-assisted"):ti,ab,kw OR ("surgical procedure, laparoscopic"):ti,ab,kw

#9 (#7) OR (#8)

#10 (#3) AND (#6) AND (#9)

**2. The terms of EMBASE**

#1 'rectum tumor'/exp

#2 'mass, rectum':ab,ti OR 'neoplasma recti':ab,ti OR 'pararectal tumor':ab,ti OR 'pararectal tumour':ab,ti OR 'rectal mass':ab,ti OR 'rectal neoplasm':ab,ti OR 'rectal neoplasms':ab,ti OR 'rectal tumor':ab,ti OR 'rectal tumour':ab,ti OR 'rectum mass':ab,ti OR 'rectum neoplasm':ab,ti OR 'rectum tumour':ab,ti OR 'retrorectal tumor':ab,ti OR 'retrorectal tumour':ab,ti OR 'tumor recti':ab,ti OR 'tumour recti':ab,ti

#3 (#1) OR (#2)

#4 'ileostomy'/exp

#5 'ileostomy relocation':ab,ti OR relocation:ab,ti OR ileostomy:ab,ti’

#6 (#4) OR (#5)

#7 'laparoscopy'/exp

#8 'laparoscopy, video':ab,ti OR 'pelvic endoscopy':ab,ti OR peritoneoscopy:ab,ti OR 'video laparoscopy':ab,ti OR videolaparoscopy:ab,ti

#9 (#7) OR (#8)

#10 (#3) AND (#6) AND (#9)

**3. The terms of Web of Science date base**

#1 Topic:(Rectal neoplasms) OR Topic:(Neoplasm, Rectal) OR Topic:(Rectal Neoplasm] OR Topic:(Rectum Neoplasm) OR Topic:(Neoplasm, Rectum) OR Topic:(Rectum Neoplasm) OR Topic:(Rectal Tumors) OR Topic:(Rectal Tumor) OR Topic:(Tumor, Rectal) OR Topic: (Neoplasms, Rectal) OR Topic:(Cancer of Rectum) OR Topic:(Rectum Cancers) OR Topic:(rectal Cancer) OR Topic:(Cancer, Rectal) OR Topic:(Rectal Cancers) OR Topic:(Rectum Cancer) OR Topic:(Cancer, Rectum) OR Topic:(Cancer of the Rectum)

#2 Topic:(lleostomy) OR Topic:(lleostomies) OR Topic:(Tube lleostomy) OR Topic:(lleostomies, Tube) OR Topic:(lleostomy, Tube) OR Topic:(Tube lleostomies) OR Topic:(incontinent lleostomy) OR Topic:(eostomies, Incontinent) OR Topic:(leostomy, Incontinent) OR Topic:(incontinent lleostomies) OR Topic:(Loop lleostomy) OR Topic:(lleostomies, Loop) OR Topic:(lleostomy, Loop) OR Topic:(Loop lleostomies) OR Topic:(Continent lleostomy) OR Topic:(Continent lleostomies) OR Topic:(lleostomies, Continent) OR Topic:(lleostomy, Continent)

#3 Topic:(Laparoscopy) OR Topic:(Laparoscoples) OR Topic:(Celioscopy) OR Topic:(Celloscople) OR Topic:(Pertoneoscopy) OR Topic: (Peritoneoscopies) OR Topic:(Surgical Procedures) OR Topic:(Laparoscopic taparoscopic) OR Topic:(Surgical Procedure) OR Topic:(Procedure, Laparoscopic Surgical) OR Topic:(Procedures, Laparoscopic Surgical) OR Topic:(Surgery, Laparoscoplc) OR Topic:(Laparoscoplc Surgica Procedures) OR Topic:(Laparoscopic Surgery) OR Topic:(Laparoscopic Surgeries) OR Topic:(Surgeries, Laparoscopic)OR Topic:(Laparoscopic Assisted Surgery) OR Topic:(Laparoscopic Assisted Surgeries) OR Topic:(Surgeries, Laparoscopic Assisted) OR Topic:(Surgery, Laparoscopic Assisted) OR Topic:(Surgical Procedure, Laparoscopic)

#4 (#1) AND (#2) AND (#3)

**4. The terms of Pubmed date base**

#1 ("Rectal Neoplasms"[Mesh]) OR (Neoplasm, Rectal[Title/Abstract])) OR (Rectal Neoplasm[Title/Abstract])) OR (Rectum Neoplasms[Title/Abstract])) OR (Rectal Tumors[Title/Abstract])) OR (Rectal Tumor[Title/Abstract])) OR (Tumor, Rectal[Title/Abstract])) OR (Neoplasms, Rectal[Title/Abstract])) OR (Cancer of Rectum[Title/Abstract])) OR (Rectum Cancers[Title/Abstract])) OR (Rectal Cancer[Title/Abstract])) OR (Cancer, Rectal[Title/Abstract])) OR (Rectal Cancers[Title/Abstract])) OR (Rectum Cancer[Title/Abstract])) OR (Cancer, Rectum[Title/Abstract])) OR (Cancer of the Rectum[Title/Abstract]))

#2 ("Ileostomy"[Mesh]) OR (Ileostomies[Title/Abstract])) OR (Tube Ileostomy[Title/Abstract])) OR (Ileostomies, Tube[Title/Abstract])) OR (Ileostomy, Tube[Title/Abstract])) OR (Tube Ileostomies[Title/Abstract])) OR (Incontinent Ileostomy[Title/Abstract])) OR (Ileostomies, Incontinent[Title/Abstract])) OR (Ileostomy, Incontinent[Title/Abstract])) OR (Incontinent Ileostomie[Title/Abstract])) OR (Loop Ileostomy[Title/Abstract])) OR (Ileostomies, Loop[Title/Abstract])) OR (Ileostomy, Loop[Title/Abstract])) OR (Loop Ileostomies[Title/Abstract])) OR (Continent Ileostomy[Title/Abstract])) OR (Continent Ileostomies[Title/Abstract])) OR (Ileostomies, Continent[Title/Abstract])) OR (Ileostomy, Continent[Title/Abstract])

#3 ("Laparoscopy"[Mesh]) OR (Laparoscopies[Title/Abstract])) OR (Celioscopy[Title/Abstract])) OR (Celioscopies[Title/Abstract])) OR (Peritoneoscopy[Title/Abstract])) OR (Peritoneoscopies[Title/Abstract])) OR (Surgical Procedures, Laparoscopic[Title/Abstract])) OR (Laparoscopic Surgical Procedure[Title/Abstract])) OR (Procedure, Laparoscopic Surgical[Title/Abstract])) OR (Procedures, Laparoscopic Surgical[Title/Abstract])) OR (Surgery, Laparoscopic[Title/Abstract])) OR (Laparoscopic Surgical Procedures[Title/Abstract])) OR (Laparoscopic Surgery[Title/Abstract])) OR (Laparoscopic Surgeries[Title/Abstract])) OR (Surgeries, Laparoscopic[Title/Abstract])) OR (Laparoscopic Assisted Surgery[Title/Abstract])) OR (Laparoscopic Assisted Surgeries[Title/Abstract])) OR (Surgeries, Laparoscopic Assisted[Title/Abstract])) OR (Surgery, Laparoscopic Assisted[Title/Abstract])) OR (Surgical Procedure, Laparoscopic[Title/Abstract])

#4 (#1) AND (#2) AND (#3)
